# HIV-1-Associated Neurocognitive Disorders: Is HLA-C Binding Stability to β_2_-Microglobulin a Missing Piece of the Pathogenetic Puzzle?

**DOI:** 10.3389/fneur.2018.00791

**Published:** 2018-09-21

**Authors:** Donato Zipeto, Michela Serena, Simona Mutascio, Francesca Parolini, Erica Diani, Elisabetta Guizzardi, Valentina Muraro, Emanuela Lattuada, Sebastiano Rizzardo, Marina Malena, Massimiliano Lanzafame, Giovanni Malerba, Maria Grazia Romanelli, Stefano Tamburin, Davide Gibellini

**Affiliations:** ^1^Department of Neurosciences, Biomedicine and Movement Sciences, University of Verona, Verona, Italy; ^2^Department of Diagnostics and Public Health, University of Verona, Verona, Italy; ^3^Service of Transfusion Medicine, AOUI, Verona, Italy; ^4^U.O.C. Infectious Diseases, AOUI, Verona, Italy; ^5^U.O.S. Infectious Diseases, AULSS 9 Scaligera, Verona, Italy

**Keywords:** HIV, AIDS, HLA, AIDS dementia complex (ADC), HIV-associated neurocognitive disorders (HAND), β_2_-microglobulin (β_2_m)

## Abstract

AIDS dementia complex (ADC) and HIV-associated neurocognitive disorders (HAND) are complications of HIV-1 infection. Viral infections are risk factors for the development of neurodegenerative disorders. Aging is associated with low-grade inflammation in the brain, i.e., the inflammaging. The molecular mechanisms linking immunosenescence, inflammaging and the pathogenesis of neurodegenerative disorders, such as Alzheimer's disease (AD) and Parkinson's disease, are largely unknown. ADC and HAND share some pathological features with AD and may offer some hints on the relationship between viral infections, neuroinflammation, and neurodegeneration. β_2_-microglobulin (β_2_m) is an important pro-aging factor that interferes with neurogenesis and worsens cognitive functions. Several studies published in the 80–90s reported high levels of β_2_m in the cerebrospinal fluid of patients with ADC. High levels of β_2_m have also been detected in AD. Inflammatory diseases in elderly people are associated with polymorphisms of the MHC-I locus encoding HLA molecules that, by associating with β_2_m, contribute to cellular immunity. We recently reported that HLA-C, no longer associated with β_2_m, is incorporated into HIV-1 virions, determining an increase in viral infectivity. We also documented the presence of HLA-C variants more or less stably linked to β_2_m. These observations led us to hypothesize that some variants of HLA-C, in the presence of viral infections, could determine a greater release and accumulation of β_2_m, which in turn, may be involved in triggering and/or sustaining neuroinflammation. ADC is the most severe form of HAND. To explore the role of HLA-C in ADC pathogenesis, we analyzed the frequency of HLA-C variants with unstable binding to β_2_m in a group of patients with ADC. We found a higher frequency of unstable HLA-C alleles in ADC patients, and none of them was harboring stable HLA-C alleles in homozygosis. Our data suggest that the role of HLA-C variants in ADC/HAND pathogenesis deserves further studies. If confirmed in a larger number of samples, this finding may have practical implication for a personalized medicine approach and for developing new therapies to prevent HAND. The exploration of HLA-C variants as risk factors for AD and other neurodegenerative disorders may be a promising field of study.

## Introduction

Neurovirulence is detectable in patients infected with human immunodeficiency virus type 1 (HIV-1) both in the early (i.e., acute infection) and the later stages of the disease. During the acute infection, 25–50% of HIV-1 patients show neurological symptoms within 3–6 weeks after infection whereas central nervous system (CNS) complications appear, to a variable extent, during the course of HIV-1 infection (1). In early studies on HIV-1 neurovirulence, the neurological syndromes were well described, and the most severe CNS form was defined as AIDS dementia complex (ADC) (2).

The cerebral complications of HIV-1 infection, which include disturbances of cognitive, behavioral, motor and autonomic functions, still represent an issue in everyday practice (3). The old concept of ADC (2, 4), a condition not commonly encountered today in Western countries because of the wide use of combination antiretroviral treatment (cART), has been replaced by the wider term HIV-associated neurocognitive disorders (HAND) (3, 5). HAND encompasses cognitive syndromes caused by HIV-1 itself, as opposed to opportunistic infections, and includes HIV-1-associated asymptomatic neurocognitive impairment (ANI), HIV-1-associated mild neurocognitive disorder (MND), and HIV-1-associated dementia (HAD) (5), the latter conditions corresponding to ADC.

HAD represents the most severe form of the spectrum of HIV-related CNS syndromes. Risk factors for HAD were reported by several studies and they include nadir CD4 count, increasing age, substance abuse, anemia, viral co-infection and viral clade subtypes (6, 7). Before the introduction of cART, the prevalence of dementia showed an annual incidence of 7% among subjects in the later stages of infection (4). Following cART introduction, HAD cases dramatically decreased, as newly diagnosed moderate to severe dementia changed from 6.6% in 1989 to 1% in 2000 (8).

The pathogenesis of CNS damage by HIV-1 is multifactorial and mediated by direct and indirect mechanisms (9). The early detection of acute meningoencephalitis in several patients indicates a rapid involvement of the brain (10). HIV-1 enters the CNS either via infected monocytes and lymphocytes or through choroid plexus infection (11, 12). The impairment of the blood-brain barrier is associated with inflammation by cytokine-driven mechanisms. In the brain, HIV-1 mainly replicates in macrophage/microglial cells thus determining the onset of chronic local inflammation. The pathological hallmark of HIV-1 infection in the brain is represented by multinucleated giant cells, which are formed by cellular syncytia of HIV-1 infected macrophages (2, 13). In addition, HIV-1 infection of astrocytes was detected in patients with HIV-1 associated encephalopathy (14). It is noteworthy that astrocyte activation and increased glial fibrillary acidic protein expression do not represent a specific response to HIV-1 infection, but are associated with other neurological conditions, such as neurodegenerative diseases (15, 16).

The persistent HIV-1 infection in the macrophages and microglia causes the releases of the phospholipid ligand PAF, glutamate, arachidonic acid, quinolinic acid, nitric oxide, and several pro-inflammatory cytokines including IL-1β, IL-6, TNFα, and TRAIL: all these factors are involved in neural damage (12, 17–23).

Persistent CNS inflammation and chronic immune activation play an important role in the pathogenesis of neurological diseases (24) where inflammatory mediators, such as neopterin, quinolinic acid, monocyte chemoattractant protein 1 and β_2_-microglobulin (β_2_m) were found to be increased (25). Importantly, these markers are also elevated in the CSF from patients with HAD suggesting that increased immune activation is related to more severe damage.

Most likely, the complexity of the pathogenetic model is not related to a one-dimensional and direct pathogenetic event, but rather to multi-dimensional and complex immunopathological processes that are governed by viral as well as by host factors (3).

### β_2_-microglobulin, HLA-C variants and HIV infection

Several old studies reported abnormally high β_2_m levels in sera of HIV-1 infected patients with high p24 concentrations and a reduced number of lymphocytes (26–28). High levels of β_2_m were also detected in the cerebrospinal fluid (CSF) of HIV-1 infected patients with ADC (29–32). HIV-infected individuals without dementia showed a consistent correlation between β_2_m levels in plasma and CSF. Contrarily in patients with dementia, CSF β_2_m level was found to be increased independently from that in plasma β_2_m levels, indicating intrathecal β_2_m production, which was proposed to be used as a marker for HIV-1 dementia (25). This association between β_2_m high levels and HIV-1 infection has never been fully explained and clarified.

β_2_m is associated with HLA proteins (A, B, or C) and a small peptide forming the major histocompatibility complex type I (MHC-I), which plays an important role in the activation and modulation of cellular immunity. The interaction between β_2_m and HLA in the MHC-I complex stabilizes the structure of β_2_m (33).

The immune complex made by HLA-C, β_2_m, and peptide expressed on the cell surface tends to dissociate and to generate a pool of free-chains molecules devoid of β_2_m. This higher HLA-C instability, compared to HLA-A and -B, is caused by the presence of a specific amino acid sequence in the groove that binds the peptide (34), which reduces its plasticity and increases its instability (35).

The binding stability of different HLA-C variants and β_2_m was documented through the analysis of the relationship between free and β_2_m associated HLA-C molecules (35). Recently, differences in specific HLA-C domains were reported to influence the peptide binding stability (36), supporting the hypothesis of the existence of HLA-C variants with a higher or a lower binding stability to β_2_m.

We previously reported that HLA-C is incorporated on HIV-1 virions increasing their infectivity (37–39), that the expression of HIV-1 Env on the cell membrane promotes the formation of HLA-C molecules as free-chain, no longer bound to β_2_m (40) and that HLA-C variants bind more or less strongly to β_2_m. We showed that some HLA-C variants may be predominantly associated with β_2_m on the cell membrane, while other ones are predominant as free-chains, dissociated from β_2_m (41). In the same study, we reported that some unstable HLA-C variants, such as C^*^03 or C^*^07, are also the ones previously described to have a low expression level, while some stable variants such as C^*^02, C^*^06, C^*^12, or C^*^16, are highly transcribed and expressed (41). HLA-C expression has been associated with a different ability to control HIV-1 infection, with high HLA-C expression levels associated to a better control of HIV-1 infection, and low HLA-C expression levels associated with poor HIV-1 control and rapid progression to AIDS (42–44).

### β_2_-microglobulin: a possible pathogenetic role in hand and neurodegeneration

β2m is one of the main markers of immune activation and inflammation in CSF in HAND (25, 45, 46).

In the CNS, β_2_m is involved in the regulation of brain development and plasticity of synapses (47–49). High levels of β_2_m are potentially neurotoxic, and β_2_m reduction has a protective effect in animal models of dementia (50). β_2_m has also been reported to be a pro-aging factor impairing cognitive functions (51).

β_2_m is responsible for “dialysis-related amyloidosis,” a clinical condition that is caused by β_2_m accumulation as insoluble protein aggregate (52) in joints, bones, and muscles (53). Studies on fatal hereditary systemic amyloidosis identified a natural variant of β_2_m which shows a dramatic decrease in thermodynamic stability and a remarkable increase in aggregation propensity (54, 55).

Of interest, high CSF levels of β_2_m were detected in neurodegenerative conditions, such as Alzheimer's disease (AD) and Parkinson's disease (PD) (56–59), which are caused by protein misfolding and abnormal aggregation (60), but the potential pathogenetic significance of this finding is still unclear.

Symptomatic similarities between HAD/ADC and AD have been documented (61). It has been shown that the Apo-ε4 haplotype, a known risk factor for AD, enhances the infectivity of HIV-1 (62) and that HIV-infected patients harboring the Apo-ε4 allele have excess dementia and peripheral neuropathy (63). ApoE has been found to stabilize and enhance the deposition of β2m amyloid fibrils (62, 64–67). In addition, the pathogenesis and clearance of the amyloid-β peptide (Aβ), the pathological hallmark feature of AD, may be influenced by HIV-1 infection. Enhanced amyloidosis has been reported in patients with HAND (68). Neurofibrillary tangles containing hyperphosphorylated tau protein are a pathological hallmark of AD. A significantly higher concentration of tau has been reported in brain tissues of HAD patients (68–71). The loss of neuroprotective functions of microglial cells has been suggested to contribute to the development of AD. A similar phenomenon is observed in HIV-infected patients since the virus can infect microglial cells, and loss of neuroprotection might trigger neurodegenerative process, leading to HAD (72).

After the report of patients with AD pathology and HIV-1 infection, the hypothesis that HIV-1 could create conditions ripe for AD development, and that a link between the two diseases does exist, has been considered (73). The view that ADC and AD share some pathogenetic pathways may pave the way for future studies comparing the commonalities between the two conditions.

### Inflammaging, viral infections, and neurodegenerative diseases

The term “inflammaging” characterizes a widely accepted paradigm that aging is accompanied by a low-grade chronic up-regulation of pro-inflammatory responses (74). Inflammaging is supposed to interact with processing and production of Aβ and to be important in the prodromal phase of AD (75). Chronic viral infections may contribute to inflammaging. Infections caused by cytomegalovirus (CMV) (76), herpes simplex (HSV) I (77–79), human herpes virus 6 (80), varicella zoster, Epstein-Barr, influenza, arboviruses, rabies and polyoma viruses, coxsackie and other enteroviruses, echoviruses (81, 82) have all been suspected to be associated with increased risk of developing CNS and neurodegenerative diseases, such as AD (83) and PD (84). β_2_m is involved in several viral infections, such as CMV, HSV, coxsackieviruses, echoviruses, and others (81, 82, 85–88). CMV has the ability to remove β_2_m from MHC-I molecules when it binds to cells it will infect (86–88), and to express an MHC-I homolog that binds and sequesters β_2_m (89). A very recent study reports that cell-free particles from the respiratory syncytial virus (RSV) and HSV type 1 may catalyze amyloid aggregation in the extracellular environment, suggesting a new connection between viral infections and neurodegenerative diseases (90).

We thus hypothesized that some HLA-C variants which more easily dissociate from β_2_m will have a higher proportion of free heavy chains, increasing HIV-1 infectivity and promoting β_2_m release, which in turn may contribute to chronic inflammation and may be involved in the pathogenesis of HAND. Similar phenomena may also contribute to the pathogenesis of neurodegenerative conditions such as AD and PD, potentially triggered by other infectious agents.

## Materials and methods

### Participants

With the introduction of cART for HIV-1, patients developing ADC have become rare; as a consequence, we were able to collect only a very limited number of subjects. From a database of almost 1100 HIV-infected subjects followed since 1985 by the Infectious Diseases Outpatient Clinic, Azienda Ospedaliera Universitaria Integrata Verona, we retrospectively selected patients who had an unequivocal diagnosis of ADC. Among these, only 11 were still available for blood sample collection and DNA extraction and analysis. We analyzed HLA-C variants in 11 HIV-1 infected individuals with ADC/HAD (HIV-ADC group), which was diagnosed using standardized brain imaging and neuropsychological criteria (91, 92).

This study was carried out in accordance with the recommendations of the Declaration of Helsinki. The protocol was approved by the Institutional Review Board of the University of Verona. All the patients, or their tutors in case of severe dementia, gave written informed consent to participate in the study.

The baseline characteristics of the patients (9 men, 2 women) are shown in Table 1. The patients had a median age of 50 years (interquartile range, IQR 39–58); all of them were naive to antiretroviral treatment. CD4+ T lymphocyte count and HIV RNA load were determined at ADC diagnosis. The median CD4+ T lymphocytes nadir was 50 cells/mm^3^ (IQR 26-157), while the median plasmatic zenith HIV-RNA viral load was 71350 copies/mL (IQR 22300-141000). A lumbar puncture was performed in 9 patients at the time when ADC was diagnosed, with a median CSF HIV-RNA viral load of 204000 copies/mL (IQR 70085-511500). The diagnosis of ADC was made using, in addition to CSF HIV-RNA viral load, brain MRI, mini-mental state examination (MMSE), and full neuropsychological evaluation. The most common findings observed on brain MRI were symmetric periventricular and deep white matter T2 hyperintensity and widespread cerebral atrophy. MMSE was <25 in all ADC patients. A full neuropsychological evaluation was performed in 4 patients and showed mild-to-moderate dementia. The severity of dementia in the remaining 7 patients made full neuropsychological testing difficult.

**Table 1:** Baseline characteristics of HIV-1 infected patients with AIDS dementia complex (HIV-ADC group).

**No**.	**Age range**	**Infection route**	**CDC CLASS ‘93**	**CD4+ T lymphocyte plasma nadir (cells/μL, % of lymphocytes)**	**HIV RNA load zenith (copies/ml)**	**HIV RNA CSF at ADC diagnosis (copies/ml)**	**HLA-C alleles**	**HLA-C stability**
1	46-50	BISEX	C3	111 (7.3%)	52100	204000	03/04	U/U
2	56-60	IDU	C3	50 (4%)	16250	72320	04/16	U/S
3	56-60	BISEX	C3	26 (1%)	205000	>500000	04/07	U/U
4	31-35	BISEX	C3	157 (7%)	22300	67850	03/07	U/U
5	36-40	BISEX	C3	47 (8%)	62300	187000	02/07	S/U
6	36-40	HETERO	C3	4 (1%)	98000	230000	06/07	S/U
7	36-40	HETERO	C2	286 (17.5%)	11853	3066	07/16	U/S
8	61-65	HETERO	C3	18 (9%)	267851	NA	02/18	S/U
9	56-60	IDU	C2	468 (40%)	NA	NA	04/14	U/U
10	51-55	HOMO	C3	41 (3%)	80400	523000	07/15	U/S
11	46-50	HOMO	C3	56 (4%)	141000	6000000	04/07	U/U

*BISEX, bisexual; IDU, injection drug user; HETERO, heterosexual; HOMO, homosexual; NA, not available; U, unstable HLA-C allele; S, stable HLA-C allele*.

We collected a control group of 16 HIV-infected patients (14 men, 2 women; median age: 46.5 years, IQR 35.5-51) with no neurocognitive disorders and MMSE ≥ 25 (HIV-no-ADC group), whose baseline characteristics are reported in Table 2. Gender and age did not significantly differ between patients and control groups.

**Table 2:** Baseline characteristics of the control group of HIV-infected patients without AIDS dementia complex (HIV-no-ADC group).

**No**.	**Age range**	**Infection route**	**CDC CLASS ‘93**	**CD4+ T lymphocyte plasma nadir (cells/μL, % of lymphocyte)**	**HIV RNA load zenith (copies/ml)**	**HLA-C alleles**	**HLA-C stability**
1	41-45	HETERO	A1	362 (14.1%)	4380	01/07	U/U
2	31-35	HOMO	A2	304 (15%)	200000	03/07	U/U
3	21-25	HOMO	B3	601 (39%)	>10000000	04/12	U/S
4	51-55	BISEX	A2	302 (18.2%)	32000	07/12	U/S
5	51-55	IDU	A1	328 (20%)	21000	04/12	U/S
6	31-35	HOMO	C2	268 (14%)	1328	07/12	U/S
7	56-60	HETERO	A1	221 (17%)	253061	06/12	S/S
8	36-40	HOMO	A3	60 (7.1%)	191463	07/12	U/S
9	61-65	BISEX	A1	713 (17%)	481000	04/15	U/S
10	31-35	BISEX	A3	120 (7.4%)	200000	06/07	S/U
11	46-50	HOMO	A2	300 (18.8%)	86000	06/12	S/S
12	46-50	BISEX	A2	214 (20.2%)	16900	02/12	S/S
13	46-50	HOMO	A1	294 (22%)	75200	04/07	U/U
14	56-60	HOMO	A2	302 (28%)	30400	02/16	S/S
15	36-40	HOMO	A2	391 (14%)	52900	14/15	U/S
16	46-50	HOMO	A2	330 (20%)	72000	04/07	U/U

*BISEX, bisexual; IDU, injection drug user; HETERO, heterosexual; HOMO, homosexual; U, unstable HLA-C allele; S, stable HLA-C allele*.

Patients were classified according to the CDC 1993 classification system, whereby HIV infection was divided into three clinical categories (i.e., A, B and C). Category A included the patients with asymptomatic acute (primary) infection or persistent generalized lymphadenopathy. Category C included the patients with AIDS-indicator conditions. The symptomatic patients, who could not be classified in categories A or C, were included in B one. HIV positive patients were further sub-grouped according to the absolute lymphocyte T CD4+ count (i.e., 1: >500 cells/μL; 2: 200–499 cells/μL; 3: <200 cells/μL) (93). According to the CDC 1993 classification, HIV-related neurological complications were described as HIV-related encephalopathy and associated to category C. Hence, HIV positive patients with ADC were classified in category C, as was the case for our 11 HIV-ADC patients (Table 1).

### HLA-C typing

HLA-C alleles were classified as unstable (HLA-C^*^01, ^*^03, ^*^04, ^*^07, ^*^14, ^*^17, ^*^18) or stable (C^*^02, ^*^05, ^*^06, ^*^08, ^*^12, ^*^15, ^*^16) according to previous phylogenetic, structural and densitometry findings (35, 36, 41, 94).

HLA-C typing was carried out at the HLA laboratory of tissue typing, Azienda Ospedaliera Universitaria Integrata (AOUI) of Verona. The laboratory is accredited by the European Federation of Immunogenetics (EFI) since 1999.

DNA was prepared from whole blood using the QIAamp DNA Blood Kit (Qiagen) and HLA-C phototyping was performed by PCR-SSP using the commercial kit “HLA-C SSP kit” BioRad, followed by gel electrophoresis for the detection of positive bands and the interpretation of the data (95).

### Data analysis

We compared HLA-C alleles (stable/unstable) and genotypes distribution between the ADC group and the population of northern Italy, low-resolution typing (96). Guerini and coworkers (2008) described HLA-C allele frequencies in a large Italian population cohort showing a consistent picture of their distribution in Italy. These data can be currently used as reference population for clinical and anthropological studies. Similarly, we compared the HIV-no-ADC group with the same reference population. Finally, we compared the two HIV-infected groups, i.e., HIV-ADC vs. HIV-no-ADC.

The significance of the association between groups of categorical data (stable and unstable alleles) was examined using 2 × 2 contingency tables and the Fisher's exact test, while quantitative data were examined using the Mann Whitney U test. The Fisher's exact test is conservative but guarantees type I error control for small sample sizes.

Genetic associations were conducted using a 2 × 2 contingency table of 2N case-control by allele counts, where N is the number of individuals tested. The strength of association was reported as Odds Ratio (OR) with a 95% confidence interval (CI).

When the statistical significance was tested under the hypothesis of a specific direction of association (i.e., unstable alleles are positively associated with ADC), a one-tailed test was employed. A two-tailed test was instead used when the association was explored without any expectation about the direction of the association between the tested variables. An association was deemed to be statistically significant by setting a significant level of 5%, that is all tests showing a nominal *p*-value <0.05 were considered to be statistically significant. Given the exploratory nature of this study, no correction for multiple testing was employed.

## Results

All 11 HIV-ADC subjects carried unstable HLA-C alleles in homozygosis (U/U, *n* = 5) or in heterozygosis (U/S, *n* = 6), but none of them was carrying stable alleles in homozygosis (S/S; Table 1).

Among the 16 HIV-no-ADC subjects, four carried unstable HLA-C alleles in homozygosis (U/U), 4 stable alleles in homozygosis (S/S) and eight were heterozygotes (U/S; Table 2).

The HLA-C stable/unstable allele distribution was different between the HIV-ADC group (stable 27%, unstable 73%) and the northern Italy population [stable 50%, unstable 50%; Fisher exact test, *p* = 0.029; OR 2.630 [CI: 1.112-∞]; Table 3].

**Table 3:** HLA-C alleles distribution (stable, unstable) in HIV-infected patients with ADC (HIV-ADC), HIV-infected patients without ADC (HIV-no-ADC), and in northern Italy (96).

	**HIV-ADC**	**HIV-no-ADC**	**Northern Italy**
HLA-C unstable	16	16	771
HLA-C stable	6	16	761

No differences in alleles distribution were observed when the HIV-no-ADC group (stable 50%, unstable 50%) was compared with the northern Italy population [Fisher exact test, *p* = 0.584; OR 0.098 [CI: 0.514-∞]; Table 3].

We also compared the HIV-ADC group with the HIV-no-ADC group, showing a slight difference in stable/unstable HLA-C alleles distribution [Fisher's exact test, *p* = 0.082; OR 2.618 [CI: 0.870-∞]; Table 3]. The two groups differed neither for sex (Fisher's exact test, *p* = 1.000) nor for age (Mann Whitney U test, *p* = 0.312).

The two HIV-infected groups did not differ for HIV RNA zenith level (Mann Whitney U test, *p* = 0.896), while they were different for CD4+ T lymphocyte nadir (Mann Whitney U test, *p* = 0.001). The lower CD4 count in HIV-infected patients with ADC is the consequence of the longer duration of HIV-1 infection with worsening immunodeficiency, which was frequent in the pre-cART times.

Finally, we analyzed the distribution of the different HLA-C alleles between the 3 groups.

We found interesting differences on HLA-C subtypes, in that HLA-C^*^12 appeared to be absent in the HIV-ADC group (0%) when compared either with the reference northern Italy population (17%, *p* = 0.038) and with the HIV-no-ADC group (25%, *p* = 0.016; Table 4). No difference was observed for the distribution of HLA-C^*^12 between the HIV-no-ADC group and the northern Italy population (*p* = 0.237; Table 4).

**Table 4:** HLA-C allele 12 distribution in HIV-infected patients with ADC (HIV-ADC), HIV-infected patients without ADC (HIV-no-ADC), and in northern Italy (96).

	**HIV-ADC**	**HIV-no-ADC**	**Northern Italy**
HLA-C^*^12	0	8	261
HLA-C Others	22	24	1271

## Discussion

Our results, despite being very preliminary, and derived from a small sample of patients, may have some implications for better understanding HAND/ADC pathogenesis and eventually improve the treatment of this condition.

ADC was common in patients with HIV-1 infection during disease progression in the pre-cART era (97). The introduction of cART regimens tackled the onset of ADC in these patients and reduced the likelihood and severity of HAND, indicating that the treatment of HIV-1 infection is essential to reduce neurological damage (98). At variance, the prevalence of less severe HAND syndromes is 46% of HIV-1-infected people and is expected to increase in the next years (99). Why HAND prevalence did not significantly decrease after the introduction of cART (100) is poorly understood (3).

To answer this question, we hypothesized that the development of HAND could be associated with the expression of specific unstable variants of HLA-C and the subsequent CNS release and deposition of free β_2_m, which has been reported to be neurotoxic and to deteriorate cognitive functions (50, 51). To this aim, from a large database of nearly 1100 HIV-infected subjects, we retrospectively selected patients who had an unequivocal diagnosis of ADC, the most severe form of HAND. Only 11 among them were still available for sample collection, DNA extraction, and analysis.

Our findings showed a higher frequency of unstable HLA-C alleles in HIV-1 infected patients who developed ADC in comparison to northern Italy general population. This finding argues in favor of a possible involvement of β_2_m in triggering and/or sustaining the development of neurologic complications of AIDS. In contrast, the HIV-infected population without ADC showed the same distribution than the general population of northern Italy, thus excluding the hypothesis that the higher frequency of unstable HLA-C alleles is associated with a higher susceptibility to HIV-1 infection. The present data raise the question whether patients expressing unstable HLA-C alleles in the control group did not develop ADC. We speculate that unstable alleles might predispose to ADC pathogenesis, but other viral and host factors (e.g., time of infection, HIV subtypes, immunological response) could contribute to the development of HAND/ADC. Moreover, the degree of stability vary across different alleles, and exploring single HLA-C variants may help better stratify the risk of ADC/HAND.

We observed an unexpected lower frequency of HLA-C^*^12, a stable HLA-C variant, in HIV-ADC subjects. Recent studies reported a protective role of HLA-C^*^12 in HIV-1 infection (101, 102). It would be tempting to speculate that the presence of HLA-C^*^12 might have some kind of protective effect on the risk of developing ADC in HIV-infected individuals. A study on a higher number of cases, allowing the analysis of the effect of single HLA-C alleles on the risk of developing neurological diseases in HIV-1 patients is definitely needed, to confirm this very preliminary but also very tempting observation.

HIV-1 neurovirulence could contribute to the dissociation of β_2_m from HLA-C molecules, as a consequence of the association of HIV-1 Env with unstable HLA-C variants (40). A three amino acid position in HIV-1 gp120 glycoprotein was recently reported to be associated with HAND (99).

The treatment of HAND is still is an issue in the era of cART (103, 104), as the improvement of neurocognitive symptoms to cART is variable (105), mild neurocognitive impairment is found in some patients, and factors predicting a response in single patients are unknown. Indeed, several studies demonstrated that cART is not effective on HAND in all HIV-1 positive patients (106, 107).

It would be reasonable to use antiretroviral drugs with high CNS penetration/effectiveness score, such as darunavir, abacavir or raltegravir (108, 109), to reduce the risk of HAND. However, some antiretroviral drugs are potentially neurotoxic: they may increase the production of Aβ protein by neurons and reduce its microglial phagocytosis, leading to the deposition of amyloid plaques in the CNS (68, 110). If our hypothesis is confirmed by further studies, an antiretroviral therapy with CNS-targeted drugs might be considered for patients with unstable HLA-C variants, paying attention to the possible side effects of some of them.

Effective cART suppresses HIV-1 replication and increases the immune system activity, thus determining a beneficial effect on the CNS through the reduction of HIV viral load, viral neurotoxic proteins production, and neuroinflammation. However, neuroinflammation may still be active as microglial activation in specific brain areas, including the hippocampus and temporal cortex, whereas in pre-cART era these changes were more common in the basal ganglia (111). Chronic inflammation and other immune mechanisms triggered by viral infection, rather than direct HIV-1 involvement, may thus play a key role in HAND pathogenesis.

ADC is rare nowadays due to the cART, and, despite our efforts, we were not able to recruit a larger ADC sample. We are fully aware that our data are preliminary and should be considered with caution. However, they are appealing and may pave the way for recruiting a larger population of patients through a multicenter study. If confirmed in larger populations of patients, these findings would suggest that HIV-infected patients carrying specific unstable variants of HLA-C should undergo strict monitoring of neuropsychological and neurological symptoms to promptly recognize early HAND stages. Moreover, CSF β_2_m levels might be explored as a molecular biomarker to recognize early HAND stages, given the absence of specific tests for this condition (112).

The observation we reported may be a starting point to explore whether some HLA-C variants are associated with higher risk of CNS viral infection, neuroinflammation and neurodegenerative disorders (113, 114). HLA-C^*^07, which is one of the most unstable variants (36), was found to be associated with AD (115). Similarly, very old studies associated HLA-C^*^03, another unstable HLA-C variant, to higher AD risk (116–118).

There are many features in common between HAND/ADC and AD, including clinical features that overlap between the two conditions (61). Aβ peptide biogenesis and clearance may be influenced by HIV-1 infection (119). Increased Aβ amyloid has been reported in up to 72% of HIV-1 patients with HAND (68), but also in 38% of those without HIV-1 but no cognitive symptoms (73, 119). We may speculate that shared pathogenetic mechanisms in response to neurotropic viral infections (e.g., herpesviridae) may contribute to Aβ amyloid deposition in AD patients without HIV-1 and that neuroinflammation due to β_2_m release in patients with unstable HLA-C alleles may accelerate this phenomenon.

The large number of HIV-infected patients under cART with a long-life expectancy due to a nearly complete control of viral load raises concern about their risk of cognitive dysfunction in the presence of cardiovascular risk factors (e.g., smoking, hypertension, diabetes, hypercholesterolemia) (120) that are known to increase also the risk of AD (121). The presence of unstable HLA-C variants might be explored as a possible predictor for worse cognitive performance at older age in HIV-infected patients with cardiovascular risk factors.

## Conclusions

Our very preliminary data suggests that HIV-1 infected individuals carrying unstable HLA-C allelic variants may be at higher risk of CNS complications. Specifically, we observed that alleles encoding unstable HLA-C variants were more frequent in HIV-1 infected patients who developed ADC, the most severe HAND subtype. The identification of specific variants of HLA-C as risk factors for HAND can provide a contribution both in the field of personalized medicine and for the development of new therapies, aimed at preventing and/or reducing neurocognitive damage in AIDS patients.

Given the potential commonalities between HIV-induced aging, neurocognitive decline and AD that represent a promising future hot research topic (73), our findings might be helpful for better understanding the pathogenesis, and for identifying disease-modifying therapeutic strategies to be offered to patients at higher risk of neurodegenerative disorders at presymptomatic disease stages.

## Author contributions

DZ conceived, designed supervised, and coordinated the study, conducted data analysis and took care of drafting and writing the manuscript. MS, SM, FP, and ED were involved in samples collection, extraction, and analysis. EG and VM conducted HLA-C typing. SR, EL, MM, and ML contributed to patients selection, clinical data collection, and manuscript writing. GM conducted genetic and statistical analysis. MGR helped to coordinate the study and assisting in manuscript preparation. ST and DG contributed to planning the study, to the analysis and collection of neurologic and virologic data and to manuscript drafting and writing.

### Conflict of interest statement

The authors declare that the research was conducted in the absence of any commercial or financial relationships that could be construed as a potential conflict of interest.
